# Management of Spontaneous Intracerebral Hemorrhage

**DOI:** 10.1007/s11910-017-0783-5

**Published:** 2017-09-08

**Authors:** Roland Veltkamp, Jan Purrucker

**Affiliations:** 1 Department of Stroke Medicine, Imperial College London, Charing Cross Campus, 3 East 6, London, W6 8RF UK; 2 Department of Neurology, Heidelberg University Hospital, Heidelberg, Germany

**Keywords:** Hematoma growth, Hemostatic therapy, Antihypertensive, Anticoagulation

## Abstract

**Purpose of Review:**

We review the current evidence for medical and surgical treatments of spontaneous intracerebral hemorrhage (ICH).

**Recent Findings:**

Therapy with hemostatic agents (e.g. factor VIIa and tranexamic acid) if started early after bleeding onset may reduce hematoma expansion, but their clinical effectiveness has not been shown. Rapid anticoagulation reversal with prothrombin concentrates (PCC) plus vitamin K is the first choice in vitamin K antagonist-related ICH. In ICH related to dabigatran, anticoagulation can be rapidly reversed with idarucizumab. PCC are recommended for ICH related to FXa inhibitors, whereas specific reversal agents are not yet approved. While awaiting ongoing trials studying minimally invasive approaches or hemicraniectomy, the role of surgery in ICH remains to be defined. Therapies targeting downstream molecular cascades in order to prevent secondary neuronal damage are promising, but the complexity and multi-phased nature of ICH pathophysiology is challenging. Finally, in addition to blood pressure control, antithrombotic prevention after ICH has to consider the risk of recurrent bleeding as well as the risk of ischemic events.

**Summary:**

Treatment of acute ICH remains challenging, and many promising interventions for acute ICH await further evidence from trials.

## Introduction

Spontaneous, non-traumatic intracerebral hemorrhage (ICH) accounts for 10–15% of all strokes, but its contribution to overall stroke mortality and disability is over-proportionally high [1]. Fifty-eight percent of ICH patients die within 1 year, and 2/3 of survivors remain moderately or even severely disabled [2]. Various forms of cerebral small vessel diseases underlie the majority of spontaneous ICH. Additional causes include vascular malformations, cerebral sinus vein thrombosis, tumors, vasculitis, and antithrombotic medication.

The prognosis and pathophysiology of ICH are increasingly better understood. In addition to non-modifiable prognostic patient characteristics such as age and pre-morbid disability including cognitive deficits, the clinical presentation and the size of the hematoma are crucial prognostic factors [3]. Importantly, hematoma size is dynamic particularly in the first hours after the event. About one-third of ICH patients presenting within the first 3–6 h after symptom onset will have hematoma growth which worsens outcome [4]. Hence, prevention of hematoma growth has become a key target of early ICH management. Another important, but more delayed process is the development of peri-hematomal edema which contributes substantially to the space-occupying effect of the hematoma. Compared to ischemic stroke, post-hemorrhagic edema may have different kinetics. Edema onset begins later according to some but not all reports [5, 6], and post-hemorrhagic edema resolves only after weeks. Extravasated blood components including heme, iron, and thrombin in the brain trigger several toxic and inflammatory cascades that contribute substantially to edema and secondary brain cell damage.

With better insights into the pathophysiology of acute ICH, medical and surgical treatment options for ICH have been under intense investigation over the last 10 years. There is good evidence that, similar to ischemic stroke, management of ICH patients on specialized multidisciplinary stroke units or neuro-critical care units improves outcome [7]. In contrast, current treatment guidelines for ICH reflect that many aspects of ICH management have remained vague even after publication of large clinical trials. In some areas, evidence is mounting that interventions are not only ineffective but rather harmful, and should hence be avoided.

In the present review, we will summarize recent evidence regarding hemostatic, antihypertensive, surgical, and neuroprotective management of ICH. We also describe ongoing efforts to tackle the challenges of ICH management and provide an outlook to where the field is heading.

## Prevention of Hematoma Growth

Hematoma growth occurs predominantly during the first few hours after ICH onset, but delayed hematoma growth can happen particularly in anticoagulated patients (see subsequent texts). The mechanisms underlying hematoma growth are not entirely clear. Consistent with a proposal originally made by Miller-Fisher based on his histopathological findings, the initial hematoma leads to twisting of the surrounding tissues which predisposes other potentially diseased microvessels to tear successively and thereby produce a “hemorrhagic avalanche” [8, 9]. Interestingly, the “avalanche” hypothesis is supported by an association between the apolipoprotein E (APOE) epsilon 2 allele, itself linked to an increased fragility of vessel walls, and hematoma expansion in lobar ICH [10]. Additional therapeutically amenable forces predisposing to continued bleeding include a raised arterial blood pressure and a deficiency of hemostatic factors.

### Blood Pressure

Lowering arterial blood pressure in acute ICH has been studied in several trials. Smaller phase 2 trials suggested that lowering blood pressure below 140 mmHg within 1 h in ICH patients admitted within 6 h after symptom onset is safe and may improve outcome and reduce the risk of hematoma expansion [11, 12]. Subsequently, two large phase 3 RCTs were performed. The INTERACT-2 trial examined the effect of intensive systolic blood pressure lowering (< 140 mmHg) compared to standard treatment (< 180 mmHg) on death and major disability (modified Rankin Scale (mRS) 3–6) [13•]. One hour after randomization, the blood pressure target was achieved in only one-third of the patients in the intensive treatment group. At 3 months, there was a strong trend for the primary endpoint in favor of intensive blood pressure lowering, and there was a statistically significant effect on the ordinal analysis of the modified Rankin scale score, a secondary endpoint. In contrast, neither hematoma growth (35.1 vs. 33.1%) nor mortality (11.9 vs. 12%) differed significantly between groups [13•]. As only a minority of patients suffered anticoagulation-related ICH (2.9%), results cannot be generalized to this important ICH subgroup. Based on the INTERACT-2 trial, the current AHA/ASA guideline states that blood pressure lowering to 140 mmHg or lower is safe and can be effective to improve functional outcome [14].

In the multicenter ATACH2-trial, ICH patients with a Glasgow Coma Scale of 5 or more were randomized within a 4.5 h time window to an intensive systolic blood pressure target of 110 to 139 mmHg or a “standard” target of 140 to 179 mmHg [15•]. The primary endpoint of the trial was the dichotomized mRS score (0–3 vs. 4–6). Besides a pre-specified rescue-therapy if systolic blood pressure exceeded the target for 30 min, only nicardipine infusion was allowed to manage blood pressure [15•]. The trial was stopped due to futility after enrolment of 1000 patients (intensive lowering mRS 4–6; 38.7 vs. 37.7% in standard treatment) [15•]. There was no difference in the rate of hematoma expansion [15•]. The mean standard treatment group minimum systolic blood pressure was 141 mmHg after 2 h, compared to 129 mmHg in the intensive treatment group. Thus, it is likely that the neutral results of the trial were a result of the already very good blood pressure management in the group receiving standard treatment. Notably, renal adverse events were more common in the intensive treatment group which may argue for less aggressive blood pressure lowering.

Blood pressure lowering is likely to be relevant also in anticoagulant-related ICH (see subsequent texts). In 853 patients with vitamin K antagonist (VKA)-related ICH, an increased rate of hematoma expansion in patients with systolic blood pressure >=160 mmHg (52.4%) compared to < 160 mmHg (33.1%), assessed 4 h after admission, was found [16•]. Blood pressure lowering to < 160 mmHg was associated with a reduction of in-hospital mortality.

### Hemostatic Factors

Stopping the expanding bleeding by enhancing blood clot formation at the site around the vessel rupture is an attractive therapeutic concept. Recombinant factor VIIa is a particularly potent agent as it is activated by tissue factor, a protein that is abundant in brain and exposed upon vascular injury. The initial phase 2 NovoSeven trial found a substantial benefit of factor VIIa over placebo in terms of hematoma growth and clinical outcome when administered within 4 h after symptom onset [17]. The subsequent FAST trial confirmed the preventive effect of rFVIIa (80 μg/kg body weight) on hematoma growth but failed to show a clinical benefit [18]. This lack of clinical benefit was partially driven by an excess of thromboembolic events in the rFVIIa group resulting from its prothrombotic effect [18, 19]. A potential resolution of this dilemma of promoting hemostasis and the adverse thromboembolic events may be to improve the selection of patients to the population that has the highest likelihood of benefit from hemostasis (i.e. patients at high risk of hematoma expansion). Such an indicator of an increased risk of hematoma growth may be the “spot sign” which reflects contrast extravasation on post-contrast CT scans [20]. Patients with a spot sign had a 2.6-fold higher risk of hematoma expansion in one study [21], but a recent sub-analysis of the ATACH-II trial challenges the predictive power of the spot-sign [22]. Hypodensities within an ICH on non-contrast CT were also recognized as an independent sign of hematoma expansion [23]. Potential other non-contrast CT imaging markers associated with hematoma expansion include the blend sign (i.e. hypoattenuating areas adjacent to hyperattenuation within ICH) and the swirl sign [24, 25]. The SPOTLIGHT and STOP-IT trials enrolled non-anticoagulated ICH patients presenting within 6 h after symptom onset who had a spot sign on baseline CT imaging. Both trials had slow recruitment, and the recently presented joint analysis included only 142 patients [26•]. The rate of hematoma expansion was low in the trials, and no significant difference was found between treated and untreated patients.

Another promising hemostatic agent is tranexamic acid (TXA) which promotes hemostasis by blocking plasminogen binding to fibrin. Importantly, there is some evidence from the CRASH-2 trial, a large RCT in major trauma patients, that tranexamic acid reduces mortality [27]. The CRASH-3 trial, focusing on the effects of TXA in traumatic brain injury, is ongoing. At present, several RCTs investigate the potential of tranexamic acid also in spontaneous ICH. The Tranexamic acid for hyperacute primary IntraCerebral Hemorrhage (TICH-2) trial is an RCT which will enroll up to 2400 patients with spontaneous ICH presenting within 8 h after symptom onset. Patients are randomized to receive TXA (1 g bolus followed by infusion of 1 g over 8 h) or placebo. Results are expected in 2018. The ongoing STOP-AUST trial selects ICH patients for TXA vs placebo based on the spot sign. The trial aims to enroll 100 patients, and results are expected later in 2018. Additional trials with TXA in ICH include the placebo-controlled Tranexamic Acid for Acute ICH Growth prEdicted by Spot Sign trial (TRAIGE; clinicaltrials.gov NCT02625948), the Tranexamic Acid for Spontaneous Acute Cerebral Hemorrhage Trial (TRANSACT; NCT03044184), and the Romanian Emergency Management of Spontaneous Intracerebral Hemorrhage (EsICH) study. Furthermore, the Swiss TICH-NOAC trial (NCT02866838) specifically focuses on efficacy of TXA in patients suffering NOAC-related ICH. Alternative hemostatic agents are in early-phase clinical development. In the phase 1b Pfizer ICH trial (clinicaltrials.gov, NCT02687191), the safety of a recombinant factor Xa (PF-05230907) is tested. This rFXa (FXa^I16L^) is more resistant to inactivation by plasma protease inhibitors compared to endogenous FXa [28]. While the study excludes patients with known anticoagulation, the FXa variant was shown to effectively overcome the anticoagulant effects of the FXa-inhibitor rivaroxaban as well as the direct thrombin inhibitor dabigatran in an experimental study [29]. Specific reversal agents for NOACs are discussed subsequently.

## Anticoagulant-Related ICH

Because of their much better efficacy than antiplatelets, oral anticoagulants (OAC) are increasingly used for long-term primary and secondary prevention of stroke and systemic embolism in patients with atrial fibrillation and less frequently in other indications including mechanical heart valves. The flip side of their increasing use is hemorrhagic complications. Among these, ICH is the most feared complication of long-term anticoagulation. Although extracranial major bleedings are 5- to 10-fold more frequent, ICH accounts for 58% of all bleeding-associated deaths in anticoagulated patients [30]. In population-based studies, ICH related to OAC accounts for 10–15% of all ICHs, but this proportion increases to 25% in tertiary stroke centers [31]. Anticoagulant-related ICH has a higher mortality than ICH in patients not treated with anticoagulation or antithrombotic therapy [32]. Hematoma growth occurs more frequently in patients anticoagulated with VKA, and growth is more likely to occur over a prolonged period than in non-anticoagulated patients [33]. The excess risk of hematoma expansion is the rationale for immediate anticoagulation reversal in VKA-ICH. Table 1 provides a brief overview over the most relevant non-OAC and OAC-related ICH studies analyzing baseline hematoma volume and expansion.

**Table 1:** Main characteristics of selected studies on intracerebral hemorrhage related to non-OAC and OAC

	Non-OAC				Mixed					OAC				
Reference	Kazui et al.	Brott et al.	Davis et al.	Mayer et al.	Flibotte et al.	Flaherty et al.	Cucchiara et al.	Huhtakangas et al.	Horstmann et al.	Kuramatsu et al.	Purrucker et al.	Steiner et al.	Connolly et al.	Wilson et al.
#	[76]	[77]	[4]	[18]	[33]	[78]	[79]	[80]	[31]	[16•]	[42•]	[36]	[50••]	[43]
Year	1996	1997	2006	2008	2004	2008	2008	2011	2013	2015	2016	2016	2016	2017
Study type	Retrospective analysis	Prospective observational	Pooled meta-analysis	RCT	Prospective cohort study	Retrospective cohort study	RCT substudy	Retrospective observational	Prospective observational	Retrospective cohort study (VKA)	Prospective observational (NOAC)	RCT (VKA)	Prospective observational (factor Xa antagonists incl. enoxaparin)	Pooled analysis
N	204	103	218	268 (Placebo-arm)	183 (70 Expansion analysis)	258	303 (285 Expansion analysis)	982	206 (152 Expansion analysis)	853	61	50	14 (ICH only)	91 (NOAC), 403 (VKA)
Age, mean, years	64	63	66	65	76	69	NR (OAC: 75)	69	74^b^	74	76	76	NR	Median: 80
Women, %	37.7	36.0	41.7	37.0	NR	54.3	33.9	46.2	47.6	37.9	41	38	NR	45% (NOAC), 51% (VKA)
Oral anticoagulation, no. (%)^c^	0 (0)	0 (0)	0 (0)	0 (0)	42 (23.0)	51 (19.8)	21 (6.9)	182 (18.5)	51 (24.8)	853 (100)	61 (100)	50 (100)	NR	500 (100)
Reversal therapy	–	–	(Placebo-arms)	(Placebo-arm)	VK + FFP	–	Not specified	Partly: VK + PCC	VK + PCC or FFP	VK+/− PCC + −FFP	Partly: PCC	PCC vs. FFP	Andexanet alfa	Partly PCC
Hematoma volume (mL)														
Median (IQR)	NR	NR	NR	NR	NR	NR	Non-OAC: 14.4 (7.9–30.9) OAC: 30.6 (7.4–70.1)	NR	Non-OAC 14.3 (4.9–35.7) OAC: 20.0 (8.3–48.8)	19.3 (6.9–52.8)	10.8 (4.0–30.0)	FFP: 13.2 (0.2–43.9) PCC: 13.0 (0.6–78.1)	NR	NOAC 14.4 (3.6–38.4); VKA 10.6 (4.0–27.9)
Mean (SD)	20.1 (18.0)	26 (29)	25.3 (NR)	22 (24)	NR	d	NR	Non-OAC: 29.6 (37.0) OAC: 47.8 (58.0)	Non-OAC: 26.4 (31.7) OAC: 31.5 (30.2)	NR	23.7 (31.3)	NR	NR (8/14 ≤ 10 mL; 6/14 11 to 60 mL)	NR
Hematoma expansion														
Predefined time frame analyzed	0–120 h	0–20 h	24 h	21–48 h	0–7 days	–	0–72 h	NR	24–48 h	NR	3–72 h	3 h, 24 h, 72 h	1 h, 12 h	< 72 h
Definition of significant expansion	> 12.5 mL or > 40%	≥ 33%	> 33%	–	≥ 33%	NR	≥ 33%	NR	≥ 6 mL or ≥ 33%	≥ 33%	≥ 6 mL or ≥ 33%	≥ 33% or death^f^	“Effective hemostasis: ≤ 20%; good hemostasis: > 20% ≤ 35%”	≥ 6 Ml or ≥ 33%
Proportion of patients with significant expansion	19.6%	38.0%	31.6%	NR^e^	Non-OAC 23% OAC: 54%	NR	Non-OAC: 26% OAC: 56%	NR	Non-OAC: 11.7% OAC: 12.5%	36%	38%	24 h: FFP: 60% PCC: 30%	NR (intracranial hemorrhage, effective/good hemostasis: 80% (56–94%)	NOAC 40%, VKA 34%

Modified and updated from Purrucker et al. [42•]

*NR* not reported, *RCT* randomized clinical trial, *OAC* oral anticoagulation

^a^References within the main document

^b^Median

^c^Oral anticoagulation = treatment with vitamin K antagonists, if not otherwise specified

^d^Non-OAC (International Normalized Ratio (INR) < 1.2) 13.4 mL, OAC INR 2.1–3.0 14.0 mL, OAC INR > 3.0 33.2 mL

^e^Estimated mean volume increase 26%

^f^Further secondary imaging endpoints included hematoma expansion ≥ 15% and/or death

### Vitamin K Antagonists

VKA anticoagulate via inhibiting the synthesis of vitamin K-dependent coagulation factors in the liver (i.e. factors II, VII, IX, X, protein C and S). Rapid replacement of deficient coagulation factors in case of bleeding is the preferred method of anticoagulation reversal. Substitution of vitamin K also reverses anticoagulation, but it is not suitable for immediate reversal of VKA, as measurable effects take hours to days. Prothrombin concentrates (PCC) and fresh frozen plasma (FFP) were the most frequently considered options for reversal in the past. In contrast to FFP which are stored in blood banks, PCC are readily available, do not need compatibility testing before transfusion, and can be infused over a few minutes. Infusion of FFPs requires infusion of large volumes which is time-consuming and can cause fluid overload. Recent RCTs have shown that PCC more rapidly and consistently reverse anticoagulation in patients with major bleeding [34, 35]. In the INCH trial, a phase 2 RCT enrolling 50 patients with intracranial hemorrhage, PCC also reduced the proportion of ICH patients with early hematoma growth and with early mortality attributed to hematoma growth [36••]. These findings are corroborated by the observational RETRACE study which found that risk of hematoma expansion was associated with the INR level after OAC reversal (RR 2.3; 95% CI 1.3–4.1; *p* = 0.005), but not the initial INR obtained at admission [16•]. Patients who achieved an INR < 1.3 had significantly fewer rates of hematoma expansion than those who did not (27 vs. 45%, *p* < 0.001). Time until complete reversal of anticoagulation is essential as a beneficial effect of INR normalization was only observed until approximately 4 h after admission [16•]. Although efficacy of early reversal of anticoagulation by PCC in terms of functional outcome still remains to be shown, PCC are the preferred reversal agents in VKA-ICH. Reversal of VKA anticoagulation can be accelerated if the INR is tested and monitored during reversal using a point-of-care coagulometer in ICH patients [37]. Administration of coagulation factors including PCC carries a risk of triggering thromboembolic complications. However, in a retrospective study of 205 patients with ICH, thromboembolism attributed to PCC was a rare occurrence [38]. Finally, in light of the limited half-life of coagulation factors, replacement of coagulation factors should always be accompanied by administration of vitamin K to enable hepatic synthesis of coagulation factors.

### Non-Vitamin K Antagonist Oral Anticoagulants

NOACs comprise the direct thrombin inhibitor dabigatran and the factor Xa inhibitors apixaban, edoxaban, and rivaroxaban. NOACs have at least similar efficacy and are safer than VKA in terms of major bleeding for stroke prevention in AF [39]. Importantly, they carry about a 50% lower risk of intracranial hemorrhage compared to VKA [39]. Nevertheless, if NOAC-related ICH occurs, the outcome including mortality is similar to ICH associated with VKA [40, 41]. Initial case-series suggested that the risk of hematoma growth in NOAC-related ICH was not increased compared to non-anticoagulated patients. As larger series have refuted this observation, there is a rational for reversal of anticoagulation in NOAC-ICH [42•, 43]. Rapid diagnosis of anticoagulant intensity of NOACs depends largely on coagulation tests that are not part of routine coagulation testing so far [44]. Point-of-care coagulometry for NOACs is desirable to speed decisions on reversal, and a number of devices are currently under development [45].

Reversal agents specific to dabigatran and the factor Xa inhibitors, respectively, have been developed. The only currently licensed specific reversal agent is idarucizumab, a humanized Fab fragment binding to dabigatran. Bolus injection of idarucizumab rapidly reverses the anticoagulant effect of dabigatran in healthy volunteers [46], patients with renal failure [47], and patients with acute major bleedings including ICH [48••]. There is no evidence of prothrombotic effects. However, experience with idarucizumab in dabigatran-related ICH from the REVERSE-AD study is limited so far. In a murine model of ICH, the Fab fragment reduces bleeding and mortality [49].

Factor Xa inhibitors can be rapidly reversed with an infusion of andexanet alfa, a recombinant, genetically modified FXa that has lost the pro-coagulant effect. The bolus is followed by a continuous infusion over 4 h. Dosing of andexanet alfa depends on the FXa inhibitor used and the time since last intake [50••]. In the first data set of the ANNEXA-4 trial reported in late 2016, rapid reversal was accompanied by a partial rebound of the anticoagulant effect after the end of the infusion [50••]. The compound is not licensed yet, and the ANNEXA-4 trial continues with a focus on ICH.

The third specific reversal agent, ciraparantag, is a small molecule that binds all NOACs (and some other anticoagulants) via hydrogen bonds and charge-charge interactions [51]. Infusion of ciraparantag reverses the prolonged bleeding time in healthy subjects taking different NOACs, but information from later phase clinical studies on safety and efficacy is not available yet [52]. Interestingly, PCC have some capacity to reverse NOACs as well. They are currently recommended for reversal of FXa inhibitors in major bleeding including ICH, but evidence for clinical efficacy in ICH is not available [53].

### Antiplatelets

A relevant number of patients with ICH are taking antiplatelet medication at the time of the event. Cyclooxygenase inhibitors such as aspirin and the P2Y2G inhibitors clopidogrel, prasugrel, and ticagrelor irreversibly block their targets in platelets and thereby attenuate platelet aggregation. As availability of functional platelets appears desirable in critical bleeding events such as ICH, transfusion of platelets or stimulation of von-Willebrand factor release and platelet aggregation by desmopressin have been advocated [54]. The randomized PATCH trial investigated in 190 patients under antithrombotic treatment the effect of platelet transfusion within 6 h of acute supratentorial ICH. Surprisingly, the study revealed that platelet transfusions increased mortality or dependence at 3 months (adj. OR 2.1, *p* = 0.0114) [55]. Hence, platelet transfusion should not be performed in ICH patients taking antiplatelet medication.

## Surgery

Removal of the extravascular blood in the brain is a plausible strategy in ICH for various reasons. First, reducing the size of the intracerebral hematoma reduces its space-occupying effect. Beyond reducing the physical forces, removal of blood may also reduce toxic blood components that are released from lysed blood cells such as heme or are part of the blood plasma such as thrombin. Unless the space-occupying effect is immediately life threatening, surgical procedures are usually delayed for a few hours to reduce the risk of recurrent bleeding.

A previous systematic review and meta-analysis of surgical trials suggested a beneficial effect of surgery on mortality [56]. The randomized, controlled STICH trial investigated whether early surgery with hematoma evacuation within a maximum of 4 days is beneficial compared to standard treatment. Inclusion of patients was only possible if the local neurosurgeon was uncertain about the benefits of either treatment. The study failed to confirm a benefit of surgical clot removal [57]. Subsequently, the STICH-2 trial was performed based on a subgroup analysis of the STICH trial that had suggested a benefit of surgery for patients with lobar hematoma location. However, no benefit of early hematoma evacuation (< 12 h after randomization) was shown in STICH-2 for patients with lobar ICH [58].

More recent surgical approaches towards ICH aim to be less invasive. In the MISTIE program, a catheter is introduced via stereotactic guidance into the hematoma. In addition to blood drainage, small doses of rtPA are injected into the ventricles to liquefy the hematoma. The phase 2 MISTIE trial suggested that the procedure is safe and may be beneficial [59••]. Results of the ongoing phase 3 MISTIE III trial (NCT01827046) are expected in 2018/9. A minimally invasive approach requiring only small craniotomies or burr holes to allow mechanical hematoma evacuation is currently investigated in a phase II trial (INVEST), and further studies are planned [60, 61].

Hemicraniectomy is another surgical approach that targets the space-occupying effect of the hematoma in analogy to the benefit observed in patients with malignant middle cerebral artery infarction. A small case series supported effectiveness of the approach in ICH, but further evidence from the SWITCH trial (NCT02258919) is necessary to evaluate the usefulness of the procedure.

Intraventricular hemorrhage (IVH) can occur in isolation or more frequently as part of parenchymal ICH where it is a negative prognostic factor [62]. A particular complication of IVH is blockage of the circulation of cerebrospinal fluid in the cerebral ventricles resulting in hydrocephalus. Placement of an external ventricular drain (EVD) is a standard procedure in this situation, but pure CSF drainage may not suffice if large volumes of blood obstruct the lateral ventricles or block the third and fourth ventricles. Moreover, prolonged presence of EVDs increases the risk of ventriculitis. Clot lysis can be accelerated by intraventricular injection of low doses of rt-PA. Initial studies of the CLEAR-IVH program suggested that low-dose rtPA injection in IVH is safe [63]. In the recently published CLEAR-III trial, a dose-dependently better resolution of the intraventricular clot was observed in rt-PA patients, but this failed to affect overall outcome [64••]. As post-hoc analyses suggested a benefit in patients with effective clot removal, a subsequent trial may focus on personalized dosing of the lytic based on close monitoring of clot removal in individual patients.

## Blocking Secondary Deleterious Toxic Processes

Experimental evidence strongly suggests that numerous cellular and molecular cascades are triggered by extravascular blood in the brain which contribute to hemorrhagic brain injury. Key pathophysiological processes include intracellular calcium release due to thrombin-mediated activation of protease-activated receptors and toll-like receptor 4 activation by fibrinogen and heme via microglia activation which leads to activation of transcription factors and expression of proinflammatory cytokines [65–68]. Erythrocyte damage releases iron, and particularly iron not bound to heme is associated with free radical formation, brain edema, and secondary neuronal death [69]. Deferoxamin, a potent iron chelator reduced post-ICH edema formation and improved functional outcome in experimental ICH [69]. This has been translated into the ongoing phase 2 Intracerebral Hemorrhage Deferoxamine (iDEF) trial (NCT02175225). The RCT aims to enroll 294 patients to receive either deferoxamin within 24 h of ICH onset for 3 days or placebo. The primary endpoint is “good functional outcome” (mRS 0–2) at 90 days. Results are expected in late 2018.

nflammation is now recognized as an essential component contributing to acute brain damage and repair [68]. The initial local response in the brain is triggered by release of danger-associated molecular patterns (DAMPs) from injured brain cells [65]. These DAMPs rapidly activate microglia which secrete toxic proinflammatory cytokines including IL1-beta and IL6 and chemokines that attract systemic immune cells. However, microglia and infiltrating macrophages also play important roles in clearing up blood components and potentially repair. By upregulating adhesion molecules, activated endothelial cells in the cerebral microvasculature facilitate entry of systemic immune cells including neutrophils, macrophages, and lymphocytes. Initially, these immigrating cells predominantly augment brain damage, but in later phases, their effects comprise repair and down-regulation of inflammation. Clinical studies targeting the inflammatory systems in ICH have addressed key cytokines (IL-1a), microglia, immigrating neutrophils, and T cells [68]. An advantage for translational medicine addressing immune cells in ICH is that a constantly growing toolbox of established medicines for other primary inflammatory conditions allows translation across traditional disease classes. For example, a small pilot study using repetitive MRI scanning to examine the effects of the sphingosine-1-receptor blocker fingolimod which reduces lymphocyte egress from lymphatic organs suggested a reduction in brain edema and better clinical outcome [70]. Clearly, a better understanding of the multi-phased pathophysiology of inflammation in ICH and subsequent later phase clinical trials are needed.

## Stroke Prevention after ICH

The primary concern after a devastating event such as ICH is to prevent recurrence. However, a relevant proportion of ICH patients are simultaneously at increased risk of ischemic disease including myocardial infarction and stroke. About 34% of non-anticoagulated patients are already taking a single or even dual antiplatelet agent [71]. Another 15–20% of ICH patients have an indication for long-term anticoagulation, usually because of atrial fibrillation, with 90% of them having a CHA_2_DS_2_VAS_c_ score of 2 or greater. Preventive management of these patients has to weigh the benefits as well as the risks of prescribing or withholding antithrombotic therapy. According to a systematic review examining the effect of antiplatelet therapy after ICH, there is insufficient evidence to either support or withhold antiplatelet therapy [70]. The ongoing randomized controlled REstart or STop Antithrombotic Randomized Trial (NCT02966119) will provide essential information for best management in this setting. For ICH patients with AF, evidence from meta-analyzed recent large observational trials suggests a much higher annual rate of ischemic stroke than for recurrent ICH. Anticoagulation with VKA reduces the rate of thromboembolic complications, apparently without increasing the rate of recurrent ICH significantly, and also reduced mortality [72]. This benefit may even have been greater in patients treated with NOACs. However, the underlying observational studies have methodological limitations including confounding by indication, and therefore, a randomized controlled trial is needed to resolve the uncertainty. Eventually, management in this complex situation will have to be personalized, and decision-making will include individual patient factors such as the severity of cerebral small vessel disease and cerebral microbleeds.

Hypertension is a key risk factor for ICH and ICH recurrence. In addition to absolute values of systolic and diastolic blood pressure, blood pressure variability influences the risk. Normalizing blood pressure is a main goal of preventive management. The PROGRESS trial found lowest recurrent stroke rate in patients with lowest blood pressure during follow-up [73]. Intensive BP lowering in patients with recent lacunar stroke was of greatest benefit in terms of ICH reduction in the SPS3 trial, while no reduction in ischemic stroke was found [74]. Better monitoring of blood pressure targets potentially by using telemedicine approaches is likely to improve blood pressure control and potentially stroke prevention (PROHIBIT-ICH) [75].

## Conclusions

Many promising interventions for acute ICH await further evidence from trials. Hemostatic therapy is most likely to be effective in the early hours after symptom-onset patients at high risk of hematoma growth. Rapid anticoagulation reversal is indicated in anticoagulant-related ICH, but more evidence regarding clinical efficacy is desirable. Future surgical and interventional management of ICH will focus on minimally invasive techniques. Therapies targeting downstream molecular and cellular processes are desirable, but the complexity and frequently multi-phased nature of pathophysiological processes require better understanding. When choosing appropriate secondary prevention after ICH in addition to blood pressure control, the strategy should consider prevention of recurrent bleeding as well as the frequently increased risk of thromboembolic ischemic events.
